# A Framework for Developing Health Equity Initiatives in Radiology

**DOI:** 10.1016/j.jacr.2022.12.018

**Published:** 2023-03

**Authors:** Matthew D. Bucknor, Anand K. Narayan, Lucy B. Spalluto

**Affiliations:** a Associate Chair for Wellbeing and Professional Climate, Department of Radiology and Biomedical Imaging and Executive Sponsor, Differences Matter, University of California, San Francisco, California.; b Vice Chair of Health Equity, Department of Radiology, University of Wisconsin School of Medicine and Public Health, Madison, Wisconsin.; c Chair of Health Equity, Department of Radiology, Vanderbilt University Medical Center, Vanderbilt-Ingram Cancer Center, Veterans Health Administration-Tennessee Valley Health Care System Geriatric Research, Education and Clinical Center, Nashville, Tennessee.

**Keywords:** Community engagement, data analytics, implementation science, health equity

## Abstract

**Purpose:**

In recent years, radiology departments have increasingly recognized the extent of health care disparities related to imaging and image-guided interventions. The goal of this article is to provide a framework for developing a health equity initiative in radiology and to articulate key defining factors.

**Methods:**

This article leverages the experience of three academic radiology departments and explores key principles that emerged when observing the experiences of these departments that have begun to engage in health equity–focused work.

**Results:**

A four-component framework is described for a health equity initiative in radiology consisting of (1) environmental scan and blueprint, (2) design and implementation, (3) initiative evaluation, and (4) community engagement. Key facilitators include a comprehensive environmental scan, early stakeholder engagement and consensus building, implementation science design thinking, and multitiered community engagement.

**Conclusions:**

All radiology organizations should strive to develop, pilot, and evaluate novel initiatives that promote equitable access to high-quality imaging services. Establishing systems for high-quality data collection is critical to success. An implementation science approach provides a robust framework for developing and testing novel health equity initiatives in radiology. Community engagement is critical at all stages of the health equity initiative time line.

## INTRODUCTION

In recent years, radiology departments have increasingly recognized the extent of health care disparities related to imaging and image-guided interventions. Some of these disparities, such as inequitable access to breast cancer screening, have been recognized for decades, but others, such as disparities in lung cancer screening, colorectal cancer screening, and access to advanced imaging and image-guided interventions (for example, advanced prostate cancer imaging and aneurysm diagnosis and treatment), have become more evident in recent years as the use of these imaging technologies and procedures continues to grow [1].

Initiatives to support health equity—the opportunity for all people to achieve their best health—are an essential component of a successful radiology organization. All radiology organizations should strive to develop, pilot, and evaluate novel initiatives that promote equitable access to high-quality imaging services [2]. Although several radiology departments have had noteworthy success in establishing programs in health equity, many find themselves without the essential skill sets to drive meaningful change and with limited resources for obtaining those skill sets within their respective institutions [3].

The goal of this article is to articulate key barriers and facilitators to developing health equity initiatives in radiology and to provide a framework to develop such initiatives. We will use context from three departments of radiology and the key principles that emerged when observing the experiences of these departments that have begun to engage in health equity–focused work.

## A FRAMEWORK FOR DEVELOPING HEALTH EQUITY INITIATIVES

**Fig. 1.**
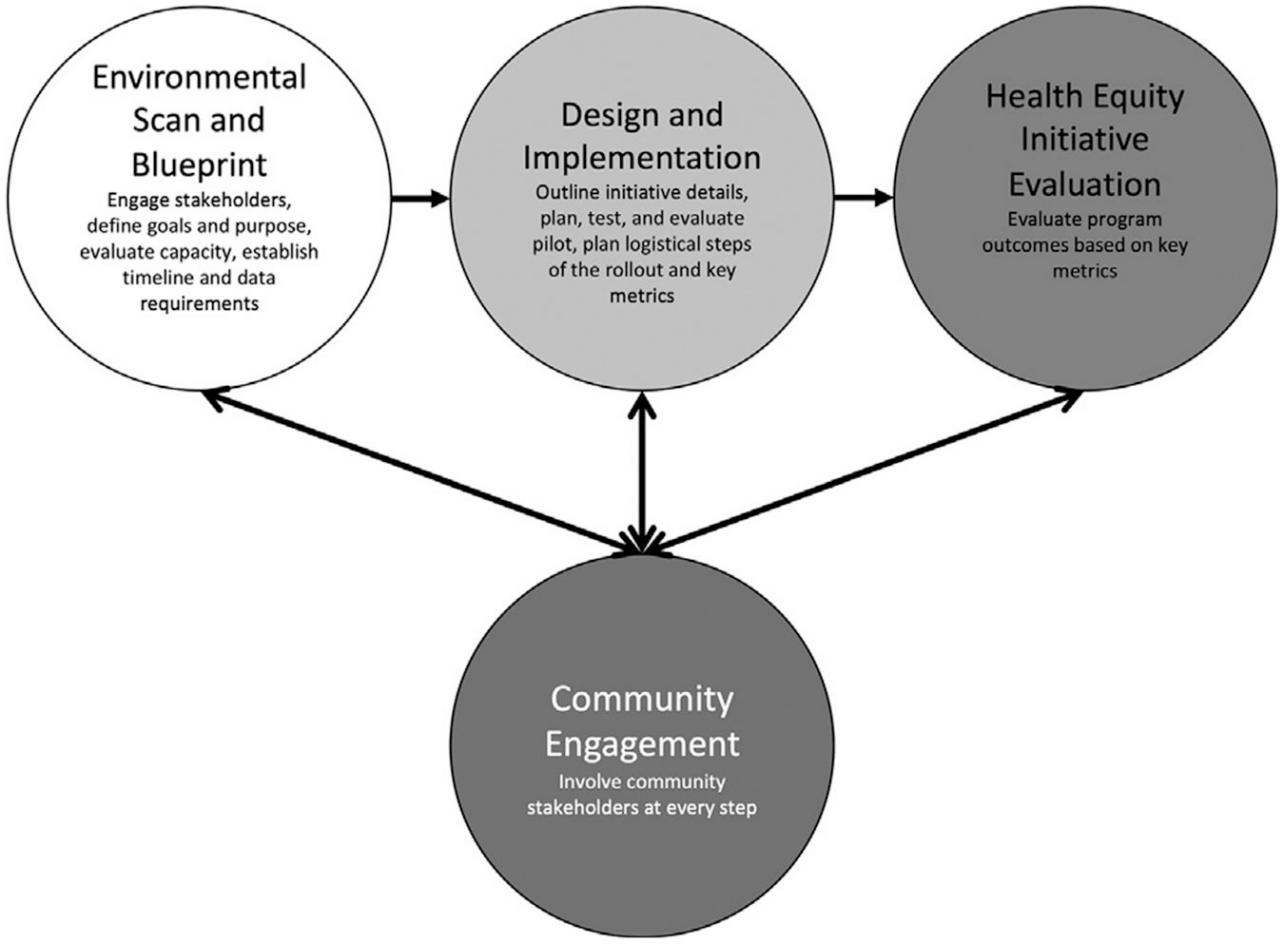
A sample framework for a four-phase departmental health equity initiative.

We propose a framework for developing health equity initiatives that can be used whether these initiatives are quality driven, research driven, or operations driven. For this framework, we propose four components (Fig. 1). In component one, a blueprint of the initiative is developed, including defining the problem to address. Goals and objectives are defined, in addition to the high-level time line, as well as core principles in addressing this problem. Also in component one, an environmental scan is performed to establish consensus regarding existing knowledge by reviewing existing literature in addition to institutional data and programs, with consultation of external organizations as needed. This environmental scan helps to better define communities at risk for each institution and can help to shape appropriate interventions. These communities may vary widely among health centers as a function of demographics. Specifically, the types of historically marginalized groups served by a particular department, for example, underrepresented minorities, urban and rural populations, immigrant populations, gender and sexual minority populations, uninsured populations, and persons with mental illness, among other groups, may vary.

In component two, the health equity initiative is designed in detail and implemented, leveraging the findings of the initial landscape assessment performed in component one. In component three, the program or initiative is evaluated through an implementation science lens. Planning for sustainability is an important component of this evaluation. *Component four* centers on community engagement and building community partnerships to ensure that the needs of the community are met. Of note, although the first three components of developing new health equity initiatives form a temporal sequence, the fourth is foundational and must be addressed throughout each of the others.

The remainder of this article focuses on challenges with quality data collection, design and evaluation of initiatives, and community engagement and partnerships, as well as strategies to overcome these challenges.

## CONDUCTING AN EFFECTIVE ENVIRONMENTAL SCAN: A CRITICAL PREREQUISITE

A critical prerequisite for development of local health equity initiatives in radiology is reliable data on imaging use and the quality of imaging services for the community served. Performing an environmental scan that makes use of both external and internal data sources is essential. Key components of environmental scans include establishing the purpose of the environmental scan, evaluating capacity for the project, creation of a time line and goal setting, stakeholder engagement, determining data requirements, analysis of results, and dissemination of results to key stakeholders [4]. Importantly, radiologists must recognize that available external data for long-studied indications such as breast and lung cancer screening are more extensive and diverse than for newer advanced imaging techniques such as molecular imaging [5]. Thus, health equity initiatives being developed for newer techniques may need to leverage examples from pre-existing work for unrelated modalities and indications. In addition to reviewing the available literature, partnering with outside institutions to share relevant work, particularly unpublished experiences, can be invaluable for refining aims and objectives and lead to novel collaborations that create a broader and more sustainable impact.

High-quality internal data sources are also necessary. Health disparities documented at other medical centers may or may not reflect local patterns of local imaging and image-guided interventions. To understand local dynamics, design appropriate and impactful initiatives, and robustly measure the outcomes of such initiatives, a consistent stream of data pertaining to health equity outcomes is necessary. Designing a system that captures internal data can present unique challenges and run counter to existing data-collecting systems.

Health equity–related data sets of interest can be divided into two broad categories: (1) the use of different imaging modalities, rates of imaging, or timing of imaging among different demographic groups with the same clinical indication and (2) differences in the quality of imaging or image-guided intervention among different demographic groups. The former requires a robust electronic medical record infrastructure that captures a wide variety of demographic variables (eg, age, gender, insurance status, preferred language, race or ethnicity, and sexual identity). Neighborhood socio-economic status may be another key driver of differential access, and typically additional steps are needed to convert patient home addresses into geospatial data, which can then be cross-labeled to established data sets of neighborhood socio-economic status [6].

Big data analytics and artificial intelligence algorithms offer great promise to target specific radiology-related health disparities and to do so most efficiently through automation [7]. However, these algorithms also have the potential to exacerbate existing disparities or create new ones and may make associations with race and other demographic factors in ways that we cannot understand or, even worse, fail to perceive, as demonstrated in a recent study in which an artificial intelligence learning model was able to predict race from medical images with high performance [8]. Early results such as these underscore the need to proceed with caution as we seek to streamline data sources for the purposes of advancing health equity.

A number of barriers and facilitators to the successful acquisition and integration of data sets into new health equity initiatives exist (Table 1). The early experiences of the University of California, San Francisco Department of Radiology and Biomedical Imaging Health Equity Council provide instructive insights. Key facilitators to robust data collection include developing broad consensus on a clear framework to create and maintain high standards for data completeness, quality, and governance [9,10]. Of note, in some cases, incomplete data may reflect an underlying disparity among patient subpopulations [11]. Therefore, departments may need to create novel prospective strategies for assessing disparities, such as questionnaires during patient appointments, to acquire the data that is needed. Some authors even advocate for collection of data from non–health data systems, such as mobile devices, social media technologies, and other online interfaces, which may be currently beyond the capacity of many radiology departments but provides an important signpost of the path forward to a truly robust data infrastructure for health equity [9].

Regarding barriers, at University of California, San Francisco, in an effort to understand the experiences of patients from different demographic backgrounds in terms of examination scheduling and turnaround times, the council initially partnered with a health systems data analytics team to develop a strategic approach. Such a collaboration, on its face, would seem straightforward, but seemingly small disagreements regarding formatting of data reporting, timing, and prioritization resulted in several months’ delay in data becoming available to the health equity council. Early involvement of department leaders, even at the initial stages of data organization, and investing in robust data analytics support based within the radiology department (as opposed to embedded within the health system) are key enabling factors, which may be easily overlooked and which strongly influence the availability of crucial data. These experiences underscore the importance of stakeholder engagement even in what may seem to be preliminary steps for a health equity initiative. Ultimately, this department’s health equity council pivoted to a strategy using data analytics support within the department, but the delay may have been avoidable with a different initial strategic approach. Successful collaborations around data are crucial for the success of health equity initiatives in radiology, and investing heavily in the early framework of data sharing is imperative.

## DESIGN AND EVALUATION OF HEALTH EQUITY INITIATIVES: LEVERAGING IMPLEMENTATION SCIENCE

An increasing number of research articles on imaging disparities have been published in recent years [1]. The vast majority of these articles document imaging disparities. Few articles describe interventions to reduce imaging disparities [12]. Implementation science provides tools to bridge known gaps in care in equitable clinical practice.

Implementation science systematically analyzes different barriers at multiple levels to develop effective strategies for implementing evidence-based practices in the real world [13]. Implementation science encourages evaluation aspects of the interventions themselves (implementation outcomes) or outcomes of these interventions (service or client outcomes) [14,15]. Implementation science methods can assess implementation, service, and client outcomes using different types of study designs. The first step requires formulating a focused implementation science question based on the extent to which a given intervention has demonstrated efficacy and effectiveness [16,17]. In 2015, the Expert Recommendations for Implementing Change project engaged implementation science and clinical practice stakeholders to participate in a modified Delphi process to publish a compilation of 73 discrete implementation strategies for the construction of multilevel implementation strategies [18].

As an example of how these approaches can be applied to a health equity initiative in radiology, prior work in breast cancer imaging provides useful lessons. People of racial or ethnic minorities develop biologically aggressive breast cancers at earlier ages and are more likely to die from breast cancer compared with White people [19]. Prior studies have concluded that early detection through improved access to breast imaging services (screening and diagnostic) can substantially reduce racial and ethnic breast cancer disparities [20]. Minority patients are more likely to experience barriers that prevent them from timely access to breast cancer screening and diagnosis [21-23]. Using an implementation science framework, we know that breast cancer screening has demonstrated efficacy and effectiveness in reducing breast cancer mortality, yet many individuals are not reaping the benefits due to barriers that prevent patients from receiving timely access to breast imaging.

To address these barriers, Wang et al designed and tested strategies to facilitate timely access to breast cancer screening and diagnosis. To improve access to screening, a “Pink Card” program was implemented that linked a physician-delivered reminder (Pink Card) that a woman is due for screening mammography during an office visit with the opportunity to undergo walk-in screening [24]. Pink Card users were more likely to be Asian or Black, Medicaid-insured, and less likely to report that English was their primary language. Additionally, racial and ethnic minorities and women who reported that English was their second language were more likely to obtain their screening mammogram on Saturdays [25]. To improve access to diagnostic imaging services, an immediate-read screening mammography program was implemented that was associated with reduced racial and ethnic disparities in same-day diagnostic imaging after abnormal screening mammograms [26].

Similarly, implementation of a same-day biopsy program was associated with the elimination of racial and ethnic disparities in biopsy timeliness [27]. Barriers to this approach included scheduling challenges associated with same-day screening and diagnostic services, limited staffing, and potential overcrowding in waiting rooms during the COVID-19 pandemic. Additionally, many facilities only perform screening mammography and require referrals to other facilities for diagnostic examinations, particularly mobile screening facilities designed to facilitate access in underserved areas. Some of these challenges were addressed with the support of strong primary care and cancer center stakeholder engagement, dedicated divisional leadership, and strict enforcement of COVID-19 safety protocols. Overall, these studies suggested that same-day screening and diagnostic services and initiatives to extend availability of breast imaging examinations outside of standard business hours increases access to breast imaging for historically underserved groups.

Although the bulk of the literature on interventions is in breast imaging [12], there are emerging initiatives in other subspecialties to improve access to imaging technologies. Chonde et al created an artificial intelligence–powered web application to enhance care delivery for patients with limited English proficiency during the COVID-19 pandemic. Using of the RadTranslate application reduced appointment variability for chest radiography performed in a predominantly Spanish-speaking community [28]. Whorms et al developed a rideshare program to assist patients encountering transportation barriers to MRI appointments. The implementation of this program was associated with improved timeliness and was more likely to be used by older patients, unemployed patients, and patients without commercial insurance [29]. These initiatives provide examples for the implementation of similar initiatives in other areas of radiology.

## COMMUNITY ENGAGEMENT AND PARTNERSHIPS

Involving the community at every stage of a health equity initiative can further bolster our ability to address imaging-related disparities [1]. Benefits include enhancing the relevance of health equity initiatives and research studies to the surrounding community, incorporating local community expertise into the development of new projects, overcoming community distrusting, and ultimately improving the health of the surrounding community [30].

Community engagement has been defined by the Centers for Disease Control and Prevention as “the process of working collaboratively with and through groups of people affiliated by geographic proximity, special interest, or similar situations to address issues affecting the well-being of those people” [31]. The Centers for Disease Control and Prevention has further defined nine principles of community engagement organized into three sections: items that should be considered before beginning engagement, what might be necessary for engagement to occur, and what might be necessary for engagement to succeed (Table 2).

Community engagement in this context exists on a spectrum, from no community involvement at all to projects and research studies that are community driven and community led [32]. A community-based participatory approach is collaborative and should actively involve community members, organizational representatives, and researchers throughout the process to address social, physical, structural, and environmental inequities [33,34]. Stakeholders can seek the input, expertise, and leadership of the community in multiple ways. Potential opportunities for community involvement include establishing a community academic partnership, working with a community advisory board, using a community engagement studio, and incorporating elements of community-based participatory research and partnership building throughout the elements of research study design and implementation [32,35-37]. Research teams should always be sure to share final study results with the community to uphold principles of transparency.

Although the benefits of a community-based participatory approach for research and outreach are evident, a number of barriers and challenges exist. These include the potential historical lack of trust and respect between institutions and community members; past and ongoing power differentials among researchers, health professionals, and the community; funding conflicts; and the time necessary to establish and maintain sustainable partnerships [33]. Radiology as a field must commit to the infrastructure and resources necessary to support community-based participatory engagement and overcome these barriers. This includes supporting researchers pursuing health equity–focused, community-engaged research through protected academic time, grants, and career development awards; developing institutional infrastructure to support community engagement (such as access to community advisory boards and community engagement studios); and establishing mechanisms for community partnerships between radiology organizations and the surrounding community.

The Breast cancer Risk Assessment—achieVing Equity (BRAVE) research study at Vanderbilt University Medical Center provides an example of a community-engaged, implementation science–driven radiology research study [38,39]. The study aims to increase the use of breast cancer risk assessment in community health centers to identify a diverse population of young women at high risk for breast cancer who may be eligible for earlier and supplemental breast cancer screening. Ultimately, the study aims to address existing breast cancer outcomes disparities by identifying earlier-stage cancers in a diverse population of young women. The study was developed in active partnership with a state and federally funded public cancer screening program, uses a community advisory board and community engagement studios to develop research study materials, and partners with numerous community health centers for study implementation. The Meharry Vanderbilt Tennessee State Cancer Partnership has helped to overcome barriers to community-engaged research by providing funding that supports both community and academic partners and access to a community advisory board. The Vanderbilt Institute for Clinical and Translational Research provides access to free community engagement studios.

## CONCLUSIONS

Health equity initiatives, whether driven by quality improvement, research, or operations, can be daunting for radiology departments that have not previously pursued this type of work. We have provided a useful framework for embarking on health equity initiatives, as well as highlighted key facilitators and barriers to quality data collection and analytics, program design and evaluation, and community engagement. Leveraging these early experiences of institutions pioneering health equity efforts provides an opportunity for others to design and implement health equity initiatives more efficiently, ultimately promoting higher-quality, more equitable care.

## Figures and Tables

**Table 1.** Key barriers, facilitators, and example of a strategic approach while performing an environmental scan, implementation, or community engagement for a health equity initiative in radiology

Phase	Barriers	Facilitators	Abbreviated Example of a Strategic Approach
Environmental scan	Lack of data or data integration	Inclusive collaborations, consensus frameworks, early involvement of department leaders	Establishing dedicated health equity data analytics support within the department
Implementation	Logistical challenges of existing workflows, underresourced health care environments	Broad stakeholder engagement, early involvement of department leaders	Creating a collaborative same-day screening and diagnostic mammography program
Community engagement	Lack of trust and respect between institutions and community members, power differentials, funding conflicts, time investment to sustain partnerships	Dedicated academic time, grants and career development awards, institutional infrastructure for engagement	Leveraging community advisory boards and community engagement studios

**Table 2.** Principles for community engagement

Overarching Community Engagement Theme	Principle of Community Engagement	Description
Items to be considered before community engagement	Be clear about the purposes and goals of the engagement efforts.	When engaging the community, individuals and organizations must be able to communicate to that community why its participation is important and worthwhile. Further, who is to be engaged, how they are to be engaged, and the goals of the engagement must be clear.
	Become knowledgeable about the community’s culture, networks, power structures, norms and values, and history.	It is important to understand and learn as much about the community as possible through qualitative and quantitative methods. It is also important to understand what the community’s current perception is of those who are initiating the engagement process.
Items necessary for community engagement to occur	Go to the community to establish relationships and trust.	Those engaging the community must adhere to the highest ethical standards to gain trust and build relationships. All partners must be actively respected from that start.
	Remember and accept that collective self-determination is the responsibility and right of all people in the community.	Internal and external forces are at play throughout the engagement process. The problems and potential solutions for these problems are defined by the community itself.
Items necessary for community engagement to be successful	Partnering with the community is necessary for change to improve health.	Equitable partnerships and transparent discussions of power are more likely to lead to the desired health outcomes. All members of the partnership must feel that they have something meaningful to contribute and something to gain.
	All aspects of community engagement must recognize and respect the diversity of the community.	Economic, educational, employment, cultural, health status, race or ethnicity, age, gender, literacy, and other aspects of diversity affect a community’s access to health care delivery and overall health. Engagement of multiple types of diverse populations can increase the success of community engagement.
	Community engagement can only be sustained by identifying and mobilizing the community’s assets and strengths and developing community capacity to take action.	Community assets include the skills, interests, and experiences of individuals and local organizations. Community resources include facilities, materials, and economic power. These assets and resources should be highlighted and leveraged for capacity building.
	Organizations and individuals seeking to effect change must be prepared to release control of actions to the community and be flexible to meet the community’s needs.	Those engaging the community must be prepared to anticipate and respond to the likely changes resulting from the community engagement process. Community engagement often drives changes in relationships and power dynamics with new coalitions networks and alliances emerging.
	Community collaboration requires long-term commitment.	The process of community engagement requires long-term commitment to build trust and lasting relationships. Long-term partnerships have the greatest capacity for making a difference in population health.

Adapted from the CDC Principles of Community Engagement.

## TAKE-HOME POINTS

- Establishing systems for high-quality data collection is critical to the success of a health equity initiative, requiring efficient integration of health systems analytics, opportunistic collection of prospective data whenever incomplete or inaccurate, and early buy-in of institutional leaders.
- An implementation science approach provides a robust framework for developing and testing novel health equity initiatives in radiology.
- Community engagement is critical at all stages of the health equity initiative time line, and managing ongoing relationships outside of the context of specific projects or research studies can influence the longitudinal success of a collaboration.
